# Patient preferences for intervention in the setting of precursor multiple myeloma

**DOI:** 10.1038/s41408-024-01161-0

**Published:** 2024-10-14

**Authors:** Catherine R. Marinac, Katelyn Downey, Jacqueline Perry, Brittany Fisher-Longden, Timothy R. Rebbeck, Urvi A. Shah, Elizabeth K. O’Donnell, Irene M. Ghobrial, Omar Nadeem, Brian L. Egleston

**Affiliations:** 1 Department of Medical Oncology, Dana-Farber Cancer Institute, Boston, MA US; 2 Centers for Early Detection and Interception, Dana-Farber Cancer Institute, Boston, MA US; 3 Zhu Family Center for Global Cancer Prevention, Harvard T.H. Chan School of Public Health, Boston, MA US; 4 Department of Medicine, Memorial Sloan Kettering Cancer Center, New York, NY US; 5 Fox Chase Cancer Center, Philadelphia, PA US

**Keywords:** Epidemiology, Disease prevention

Dear Editor,

Multiple myeloma (MM) is always preceded by monoclonal gammopathy of undetermined significance (MGUS), and smoldering multiple myeloma (SMM) [1]. These conditions confer an elevated risk of developing MM which generally ranges between one and ten percent per year, but in some individuals, the near-term risk of progression to MM is considerably higher [2]. Despite increasing interest in preventing disease progression, the cornerstone of clinical management for MGUS and SMM is watchful waiting until progression, at which point the disease is considered incurable [3, 4]. It is therefore unsurprising that even patients with a low disease burden report high levels of anxiety and a decreased quality of life under this watchful waiting paradigm [5].

There is a growing array of trials evaluating the degree to which treatment with cancer-directed therapies can prevent disease progression in patients with MM precursor conditions [6–9]. While the evidence from these studies suggests that early treatment is promising [6, 7], such interventions carry the risk of side effects that can range from fatigue and infection, to the more worrisome possibilities of clonal selection and secondary malignancy [10]. Thus, the use of treatment in the precursor disease setting has been met with controversy, and a recent survey of physicians indicates a hesitancy to treat [11]. Although non-therapeutic strategies are being investigated as alternatives (e.g., diet, metformin), their efficacy is thought to be substantially smaller than cancer-directed therapies. Information about how patients might balance the risks against benefits is lacking but is urgently needed to guide research priorities.

We developed a conjoint analysis instrument to assess different attributes that characterize patient preferences regarding intervention in individuals with MGUS and SMM. This is a survey-based statistical method that is widely used in market research to determine how people value attributes of service (e.g., cost, function, convenience) and has been increasingly applied to health care decision-making [12]. This method assumes that each treatment or service can be defined as a combination of levels of a given set of attributes. The total satisfaction or utility that an individual derives from a given intervention or service is thereby determined by the utility of each attribute [13].

The conjoint instrument described herein had four attributes consisting of two or three levels which included the chance that the intervention will prevent MM completely (25%, 50%, or 75%), monthly out-of-pocket cost ($100, $250, or $500), personal inconvenience (high or low), and risk of long-term side effects (yes or no). The attributes levels were informed by discussions with clinicians, patients, and researchers, and were selected with the intention of presenting individuals with a range of hypothetical options that would be easily understood. Participants were presented with two scenarios encompassing a combination of the four attributes and were asked, “if these were your only treatment/intervention options, which would you choose?” This experiment was repeated a total of nine times, using different combinations of attribute levels according to a balanced-overlap design. Presenting nine different choice combinations of attribute levels was done to understand how participants make tradeoffs. The full survey detailing the choice combinations with varying attribute levels are outlined in the supplementary methods.

Patient preferences were analyzed using a conditional logistic model to investigate the attributes that drive decisions about early treatment. We used cluster corrected standard errors to account for multiple repeated choice experiments within person. In addition, we used a latent profile analysis to identify latent subpopulations of patients that are defined by their attribute preferences. Finally, we used descriptive statistics and regressions to assess whether there were sociodemographic characteristics that defined the subpopulations. In sensitivity analyses, we included interaction terms in our conditional logistic regression to investigate whether prior treatment on a clinical trial moderates preferences.

Participants (*n* = 272) were 43% male, with a mean age of 64.1 (SD: 9.7) years. Most study participants were highly educated, having completed college (26.8%) or graduate school (57.2%) (Table 1). Fifty-eight percent of participants have a reported diagnosis of MGUS (vs. SMM). Forty-one (15.1%) were previously on a clinical trial testing a drug for the prevention of progression of MGUS/SMM.

**Table 1:** Descriptive Characteristic of 272 individuals with monoclonal gammopathy of undetermined significance (MGUS) and smoldering multiple myeloma (SMM) who completed survey.

**Age**, mean (SD)	64.1 (9.7)
**Sex**	
Female	156 (57.4%)
Male	116 (42.6%)
**Race**	
Non-White	15 (5.8%)
White	242 (94.2%)
**Education**	
Not college grad	41 (16.0%)
College grad	69 (26.8%)
Graduate school	147 (57.2%)
**Income**	
<$25 000	8 (3.1%)
$25 000 to $50 000	16 (6.2%)
$50 000 to $75 000	19 (7.4%)
$75 000 to $100 000	27 (10.5%)
$100 000 to $150 000	50 (19.5%)
$150 000 to $200 000	38 (14.8%)
>$200 000	71 (27.6%)
Don’t know	2 (0.8%)
Decline	26 (10.1%)
**Stage**	
MGUS	152 (58.0%)
SMM	108 (42.0%)
**How often does individual see a doctor for MGUS/SMM**	
At least every 3 months	94 (38.2%)
Every 6 months	79 (32.1%)
Annually or less often	73 (29.7%)
**Perceived risk of MM**	
Low/no risk	110 (42.1%)
Moderate risk	115 (44.1%)
High risk	36 (13.8%)
**Ever been treated on clinical trial for condition**	
Yes	41 (15.1%)
No	214 (79.0%)
I don’t know	1 (0.4%)

Logistic regression models suggest a strong preference for choosing interventions based on a higher likelihood of MM prevention (odds ratio [OR] per 10% change:1.86, 95% confidence interval [CI]: 1.72, 2.00) and avoidance of side effects (OR: 0.10, 95%CI: 0.08, 0.13) (Supplement Table 1). Higher monthly cost was also associated with a lower likelihood of selecting an intervention, although the magnitude of effect was relatively small compared to what was observed for the attributes of MM prevention and avoidance of side effects (OR per $100 change: 0.86, 95% CI: 0.80, 0.92). More personal inconvenience was also associated with a moderately reduced likelihood of selecting a particular intervention (OR: 0.62, 95% CI: 0.50, 0.76). In interaction models, there was a significant interaction between precursor stage and the effect of risk reduction on choices, whereby those in the SMM group had a stronger preference for risk reduction (OR: 2.15, 95% CI: 1.88, 2.45) than those with MGUS (OR: 1.72, 95% CI: 1.57, 1. 89); however no other attributes demonstrated significant interactions. Finally, clinical trial participation modified the effect of a reduction in MM risk on participant choice but was not a significant effect modifier of any other attribute (Supplementary Table 2).

We identified three latent profiles: the first profile defines individuals who predominantly value avoidance of side effects, the second profile characterizes individuals who value keeping costs low, and the third profile of individuals values MM prevention above other attributes (Supplementary Table 3). We assessed the degree to which various sociodemographic characteristics defined the latent profile subpopulations (Table 2). Participants in our sample predominantly prioritized preventing MM (45% in profile 3) and avoiding side effects (42% in profile 1). We found that mean age was similar across latent profiles (*p* = 0.4). A higher proportion of individuals in profile two (group prioritizing cost) did not complete college. A slightly higher proportion of individuals with SMM were grouped into the category of patients that prioritized MM risk reduction compared to the group that prioritized avoiding side effects, but the percentages were not markedly different.

**Table 2:** Characteristics of Latent Profiles.

	Profile 1: Avoid side effects	Profile 2: Low Cost	Profile 3: Prevent Myeloma	*P*-value
**Estimated proportion in group**	42%	13%	45%	
**Age**, mean (SD)	63.9 (9.7)	63.8 (9.6)	64.4 (9.6)	0.38
**Sex**				0.02
Female	59.1%	67.1%	55.9%	
Male	40.9%	32.9%	44.1%	
**Race**				0.06
Non-White	6.7%	12.0%	6.8%	
White	93.3%	88.0%	93.2%	
**Education**				0.002
Not college grad	16.1%	21.2%	14.4%	
College grad	25.1%	36.1%	26.1%	
Graduate school	58.8%	42.7%	59.5%	
**Income**				<0.001
<100 000	29.0%	51.3%	26.4%	
≥100 000	71.0%	48.7%	73.6%	
**Numeracy**, mean (SD)	4.7 (0.8)	4.6 (0.8)	4.7 (0.8)	0.03
**Perceived Risk of Multiple Myeloma**				0.03
Low/no risk	44.3%	47.0%	38.7%	
Moderate risk	42.2%	39.9%	46.4%	
High risk	13.5%	13.1%	14.9%	
**Ever been treated on clinical trial for condition**				<0.001
Yes	20.9%	14.2%	22.1%	
No	78.6%	85.8%	77.6%	
I don’t know	0.5%	0.0%	0.4%	
**Disease Burden**				0.02
MGUS	58.1%	67.0%	55.5%	
SMM	41.9%	33.0%	44.5%	

Participant characteristics are weighted to adjust for the uncertainty in assignment into a given latent profile.

Overall, this study found an overarching preference for interventions that conferred a higher likelihood of preventing MM as well as low side effect profile, which are two attributes that are difficult to achieve simultaneously in any therapeutic strategy. The most active agents for MM that are being studied in the precursor space include anti-CD38 monoclonal antibody combinations and also T-cell redirecting immunotherapies. While these agents have demonstrated high levels of efficacy, the associated toxicities must be balanced with quality of life, particularly in patients with an asymptomatic disease state. While data from the current study reaffirm that there is a sizable proportion of patients that will seek higher-risk interventional strategies with cancer-directed therapies, it also suggests an opportunity for expanding investigations of milder and potentially non-therapeutic options for the large group of patients that are most concerned about side effects.

Interestingly, the choice of efficacious intervention vs low side effect profile was not strongly related to factors that a clinician might consider as key predictors of intervention choices, such as age or perceived risk of developing MM. A proportion of low-risk patients prioritized MM risk reduction over minimizing side effects and cost, while some high-risk patients were risk-averse and prioritized reducing side effects, which is an observation that has been documented in numerous other disease setting [14, 15]. These findings reinforce the observation that there are diverse preferences among this patient population that should be considered when clinical decisions about treatment are made.

Several limitations from this study should be noted. First, recruitment and survey administration were performed online in a population of participants that are already engaged in clinical research. Thus, our sample may not accurately reflect the broader population in the US that meets our eligibility criteria. Along those lines, we note a limited representation of participants that have a low socioeconomic status, as well as low percentage of racial and ethnic minorities. Additionally, precursor disease status was self-reported, and we did not have recent medical records available to calculate more refined risk strata within precursor subgroups of MGUS and SMM. Finally, our experiment consisted of hypothetical scenarios that did not identify interventional strategies. We do not know if participants would have responded with similar preference if choices reflected real world clinical decisions, and we did not consider all variables that may be relevant to clinical decision making, such as short-term side effects, as additional variables would increase the length and complexity of the survey. Future studies may consider the degree to which short-term risks impact hypothetical decision making in this patient population and broad preference for active surveillance vs. intervention.

While there has been tremendous progress in the therapeutic options for patients with precursor disease, the long-term benefits of treatment remain an area of continued investigation, and no option to date has proven to be curative. Findings from the current study suggest that patients with MGUS and SMM have diverse risk-tolerances and preferences for intervention. Thus, a varied range of interventions will be needed meet the preferences of this unique precursor patient population.

## Supplementary information

Supplementary Information

## References

[1] Kyle RA, Larson DR, Therneau TM, Dispenzieri A, Kumar S, Cerhan JR, et al. Long-Term Follow-up of Monoclonal Gammopathy of Undetermined Significance. N Engl J Med. 2018;378:241–9. 29342381 10.1056/NEJMoa1709974 PMC5852672

[2] Landgren O. Monoclonal gammopathy of undetermined significance and smoldering multiple myeloma: biological insights and early treatment strategies. Hematol Am Soc Hematol Educ Program. 2013;2013:478–87. 10.1182/asheducation-2013.1.478 24319222

[3] Kyle RA, Rajkumar SV. Management of monoclonal gammopathy of undetermined significance (MGUS) and smoldering multiple myeloma (SMM). Oncology (Williston Park). 2011;25:578–86. 21888255 PMC3923465

[4] Ghobrial IM, Landgren O. How I treat smoldering multiple myeloma. Blood. 2014;124:3380–8. 25298034 10.1182/blood-2014-08-551549 PMC4246036

[5] Murphy B, McShane CM, Santin O, Treanor C, Byrne B, Donnelly M, et al. Patient’s perspectives of living with a precancerous condition: Monoclonal gammopathy of undetermined significance (MGUS). Eur J Oncol Nurs. 2021;51:101901. 33503552 10.1016/j.ejon.2021.101901

[6] Mateos MV, Hernandez MT, Giraldo P, de la Rubia J, de Arriba F, Lopez Corral L, et al. Lenalidomide plus dexamethasone for high-risk smoldering multiple myeloma. N. Engl J Med. 2013;369:438–47. 23902483 10.1056/NEJMoa1300439

[7] Lonial S, Jacobus S, Fonseca R, Weiss M, Kumar S, Orlowski RZ. et al. Randomized Trial of Lenalidomide Versus Observation in Smoldering Multiple Myeloma. J Clin Oncol. 2020;38:1126–37. 31652094 10.1200/JCO.19.01740 PMC7145586

[8] Korde N, Roschewski M, Zingone A, Kwok M, Manasanch EE, Bhutani M, et al. Treatment With Carfilzomib-Lenalidomide-Dexamethasone With Lenalidomide Extension in Patients With Smoldering or Newly Diagnosed Multiple Myeloma. JAMA Oncol. 2015;1:746–54. 26181891 10.1001/jamaoncol.2015.2010 PMC6662597

[9] Kazandjian D, Hill E, Dew A, Morrison C, Roswarski J, Korde N, et al. Carfilzomib, Lenalidomide, and Dexamethasone Followed by Lenalidomide Maintenance for Prevention of Symptomatic Multiple Myeloma in Patients With High-risk Smoldering Myeloma: A Phase 2 Nonrandomized Controlled Trial. JAMA Oncol. 2021;7:1678–85. 34529025 10.1001/jamaoncol.2021.3971 PMC8446896

[10] Vaxman I, Gertz MA. How I approach smoldering multiple myeloma. Blood. 2022;140:828–38. 35576526 10.1182/blood.2021011670 PMC9412010

[11] Mohyuddin GR, Chakraborty R, Cliff ERS, Derman BA. Clinician preferences on treatment of smoldering myeloma: a cross-sectional survey. EClinicalMed. 2023;65:102272. 10.1016/j.eclinm.2023.102272 PMC10689285 38046471

[12] Gelhorn H, Ross MM, Kansal AR, Fung ET, Seiden MV, Krucien N, et al. Patient Preferences for Multi-Cancer Early Detection (MCED) Screening Tests. Patient. 2023;16:43–56. 35844011 10.1007/s40271-022-00589-5 PMC9813037

[13] Ryan M, Farrar S. Using conjoint analysis to elicit preferences for health care. Brit Med J. 2000;320:1530–3. 10834905 10.1136/bmj.320.7248.1530 PMC1118112

[14] Fox RJ, Cosenza C, Cripps L, Ford P, Mercer M, Natarajan S, et al. A survey of risk tolerance to multiple sclerosis therapies. Neurology. 2019;92:e1634–e42. 30867272 10.1212/WNL.0000000000007245 PMC6448448

[15] Peres J. Why is breast cancer chemoprevention such a hard sell? J Natl Cancer Inst. 2014;106:dju139 24815870 10.1093/jnci/dju139 PMC4677265

