# Publisher Correction: Patient preferences for intervention in the setting of precursor multiple myeloma

**DOI:** 10.1038/s41408-024-01174-9

**Published:** 2024-10-21

**Authors:** Catherine R. Marinac, Katelyn Downey, Jacqueline Perry, Brittany Fisher-Longden, Timothy R. Rebbeck, Urvi A. Shah, Elizabeth K. O’Donnell, Irene M. Ghobrial, Omar Nadeem, Brian L. Egleston

**Affiliations:** 1 Department of Medical Oncology, Dana-Farber Cancer Institute, Boston, MA US; 2 Centers for Early Detection and Interception, Dana-Farber Cancer Institute, Boston, MA US; 3 Zhu Family Center for Global Cancer Prevention, Harvard T.H. Chan School of Public Health, Boston, MA US; 4 Department of Medicine, Memorial Sloan Kettering Cancer Center, New York, NY US; 5 Fox Chase Cancer Center, Philadelphia, PA US

**Keywords:** Epidemiology, Disease prevention

Correction to: *Blood Cancer Journal* 10.1038/s41408-024-01161-0, published online 14 October 2024

In this article the author’s name Omar Nadeem was incorrectly written as Nadeem Omar.

