# Supplementary material for: Patient preferences for intervention in the setting of precursor multiple myeloma
Source: Blood Cancer J. 2024 Oct 14;14(1):175. doi: 10.1038/s41408-024-01161-0 (PMC11473799; doi:10.1038/s41408-024-01161-0)
Supplement: Supplementary file 1 — Supplementary Information [file 41408_2024_1161_MOESM1_ESM.docx]

| **Supplement Table 1. Logistic Regression of Attributes that Drive Treatment Decisions in 272 MGUS and SMM Patients.** | | |
| --- | --- | --- |
| **Attribute** | **Odds Ratio (95% CI)** | **P-value** |
| **Prevent Myeloma (per 10% change)** | 1.86 (1.72, 2.00) | <0.001 |
| **Monthly cost (per $100 change)** | 0.86 (0.80, 0.92) | <0.001 |
| **Inconvenience** | 0.62 (0.50, 0.76) | <0.001 |
| **Risk of side effect** | 0.10 (0.08, 0.13) | <0.001 |
| Note: The attribute level preference weights are on the odds ratio scale from the conditional logistic regressions. | | |

| **Supplement Table 2. Effect of a 10% Reduction in MM Risk by Clinical Trial Participation.** | | | | | | | |
| --- | --- | --- | --- | --- | --- | --- | --- |
|  | **No clinical trial participation** | | | **Clinical trial participation** | | | **Interaction** |
|  | **N** | **OR** | **95% CI** | **N** | **OR** | **95% CI** | **P-value** |
| **Drug Regimen*** |  |  |  |  |  |  |  |
| Proteosome inhibitor | 251 | 1.83 | (1.70, 1.98) | 21 | 2.42 | (1.68, 3.48) | 0.145 |
| Immune therapies | 248 | 1.82 | (1.69, 1.97) | 24 | 2.71 | (1.79, 4.09) | 0.064 |
| Immunomodulatory | 248 | 1.82 | (1.69, 1.97) | 24 | 2.58 | (1.76, 3.77) | 0.082 |
| Other drugs not listed above | 242 | 1.81 | (1.68, 1.96) | 30 | 2.54 | (1.82, 3.56) | 0.054 |
|  |  |  |  |  |  |  |  |
| Confirmed clinical trial (any type)* | 231 | 1.79 | (1.66, 1.94) | 41 | 2.50 | (1.93, 3.25) | 0.017 |
| *Confirmed by medical record review | | | | | | | |

| **Supplement Table 3. Marginal Means of Attributes by Latent Profile Clusters.** | | | |
| --- | --- | --- | --- |
| **Prevent Multiple Myeloma, %** | -4.9% | -11.8 % | 30.5% |
| **Monthly cost, $** | $101 | -$348 | $144 |
| **Risk of Side effect (Chose No = -1 versus Chose Yes = 1)*** | -0.98 | 0.23 | 0.24 |
| * Values of 1 or -1 only assigned when side effect attributes varied among choice scenarios. The value was zero if side effects were the same between scenarios in a given experiment. | | | |

**METHODS**

***Patients***

Individuals with MGUS and SMM were enrolled from two nationwide cohort studies of precursor conditions to MM and other blood cancers: The PROMISE and PCROWD studies. PROMISE is a nationwide cohort study that prospectively screens a high-risk population to identify individuals with plasma cell dyscrasias, including MGUS and SMM;^1^ PCROWD is a nationwide, longitudinal cohort of individuals with an established precursor conditions to a range of blood cancers. Individuals from both studies were recruited by means of an invite letter and were asked to verify eligibility using a brief eligibility survey. Individuals were eligible for the current study if they were 18 years and over with a current diagnosis of MGUS or SMM and no concurrent diagnosis of another malignancy requiring active therapy. All eligible participants were sent a unique link to the full study survey, which was completed online. All study procedures were approved by the Institutional Review Board at the Dana-Farber Cancer Institute, and informed consent was implied by return of the study questionnaire.

Study invitation emails were sent to 1,186 patients from the PROMISE and PCROWD studies with a diagnosis of MGUS or SMM who were not known to have progressed to MM during follow up. A total of 504 individuals (42%) expressed initial interest in the study and completed an eligibility assessment; the remainder did not respond to the study email. Of these, 393 (78%) met eligibility criteria and received a unique link to the online study survey. From this sample, we received 272 completed surveys (69% response rate).

***Covariates***

Sociodemographic characteristic such as age, income, education, and race were self-reported on the study questionnaire. In addition, the survey queried about other factors that may impact an individual’s treatment choices, such as perceived risk of MM, numeracy, and tolerance for uncertainty. Perceived risk of MM was assessed using a cancer risk perception subscale adapted from the breast cancer literature.^2^ Numeracy, or the aptitude with probabilities, fractions and ratios, is an important component of understanding the risks and benefits of health behaviors and was assessed using the Subjective Numeracy Scale.^3^ Tolerance for uncertainty was assessed using the Intolerance of Uncertainty subscale.^4^ Finally, given the increasing number of clinical trials offered to individuals with MM precursor conditions at specialized centers and concerns that excluding those on trials may introduce bias towards a more risk-adverse patient population; we also queried patients on whether they had been previously enrolled in a clinical trial, and reviewed medical records to ascertain the treatment regimen used on the trial.

**Survey**

**We will be presenting you with a series of hypothetical situations related to managing multiple myeloma risk to see which situation you would choose.**

Each scenario will vary on several characteristics or “attributes”. The attributes we are using in this survey include:

- Chance that treatment will prevent myeloma completely
- Monthly out of pocket cost
- Personal inconvenience
- Long-term side effects

On the next few pages, we will describe each attribute in more detail.

# Attribute 1: Chance that treatment will prevent myeloma completely.


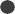
This is essentially a description of how well the treatment or intervention worked in preventing myeloma from developing. We will present this information in terms of rates, which is a “number per unit,” metric, such as “miles per hour” or “cost per pound.”


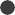
They are sometimes presented as percentages: 50% means “50 per 100.”

**Example:** If we say that the chance that treatment will prevent myeloma completely is 20% (20 per 100), then out of every 100 patients treated, 20 will never develop myeloma after treatment, and 80 could still develop myeloma at some point in their life

# Attribute 2: Monthly out of pocket cost.

# This is the cost that you would pay for the intervention after insurance

# Attribute 3: Personal inconvenience.


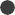
Many factors can make treatment or interventions inconvenient for patients. This can include but is not limited to time and travel for receiving treatment; it could also include short-term side effects from treatment, such as fatigue, nausea, or irritability. We expect that these inconveniences **will go away** when treatment stops.


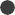
High personal inconvenience means that treatment is more likely to disrupt your daily life, whereas low personal inconvenience is less likely to disrupt your daily life.

# Attribute 4: Risk of Long-term side effects.


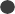
Some treatments carry risks for long-term side effects, that might not go away after treatment.


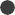
An example of a long-term side effect is peripheral neuropathy, which is caused by nerve damage and results in weakness, numbness and pain.


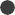
A treatment could also slightly raise your risk of developing other chronic conditions like cardiovascular disease, or slightly raise your risk of developing a different cancer. We could also consider these long-term side effects of treatment.


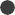
If a treatment or intervention carries a risk for long-term side effects, it means that a few patients might experience that long-term side effect. It does not mean that all patients will experience a long-term side effect.

On the next few pages, you will be given a series of nine scenarios. In each scenario, there are two options, each with a set of attributes that vary. You will be asked to choose one of the two scenarios based on your preferences.

**When responding to the hypothetical scenarios, please remember that:**

- The cases are hypothetical situations not related to your own health care
- Your answers will not be shared with your doctor
- Your own care will not be affected in any way by taking this survey There is no right or wrong answer, just choose as honestly as you can!
- Because the scenarios are randomly generated by a computer program, some of the scenarios may be unrealistic. If so, please answer to the best of your knowledge.

If these were your only treatment/intervention options, which would you choose? 1 / 9

20% (20 per 100)

$100

High

Yes

intervention_Choice1

Select

75% (75 per 100)

$500

Low

No

intervention_Choice1

Select

**Chance that treatment will prevent myeloma completely**

**Monthly out of pocket cost**

**Personal inconvenience**

**Risk of long- term side effects?**

intervention_Choice2

If these were your only treatment/intervention options, which would you choose? 2 / 9

50% (50 per 100)

$25

Low

No

intervention_Choice2

Select

75% (75 per 100)

$500

Low

Yes

intervention_Choice2

Select

**Chance that treatment will prevent myeloma completely**

**Monthly out of pocket cost**

**Personal inconvenience**

**Risk of long- term side effects?**

intervention_Choice3

If these were your only treatment/intervention options, which would you choose? 3 / 9

50% (50 per 100)

$100

High

No

intervention_Choice3

Select

20% (20 per 100)

$25

Low

No

intervention_Choice3

Select

**Chance that treatment will prevent myeloma completely**

**Monthly out of pocket cost**

**Personal inconvenience**

**Risk of long- term side effects?**

intervention_Choice4

If these were your only treatment/intervention options, which would you choose? 4 / 9

50% (50 per 100)

$25

Low

No

intervention_Choice4

Select

75% (75 per 100)

$500

High

Yes

intervention_Choice4

Select

**Chance that treatment will prevent myeloma completely**

**Monthly out of pocket cost**

**Personal inconvenience**

**Risk of long- term side effects?**

intervention_Choice5

If these were your only treatment/intervention options, which would you choose? 5 / 9

20% (20 per 100)

$100

Low

No

intervention_Choice5

Select

75% (75 per 100)

$25

Low

Yes

intervention_Choice5

Select

**Chance that treatment will prevent myeloma completely**

**Monthly out of pocket cost**

**Personal inconvenience**

**Risk of long- term side effects?**

intervention_Choice6

If these were your only treatment/intervention options, which would you choose? 6 / 9

50% (50 per 100)

$500

High

No

intervention_Choice6

Select

20% (20 per 100)

$100

Low

No

intervention_Choice6

Select

**Chance that treatment will prevent myeloma completely**

**Monthly out of pocket cost**

**Personal inconvenience**

**Risk of long- term side effects?**

intervention_Choice7

If these were your only treatment/intervention options, which would you choose? 7 / 9

50% (50 per 100)

$500

Low

No

intervention_Choice7

Select

75% (75 per 100)

$25

High

Yes

intervention_Choice7

Select

**Chance that treatment will prevent myeloma completely**

**Monthly out of pocket cost**

**Personal inconvenience**

**Risk of long- term side effects?**

intervention_Choice8

If these were your only treatment/intervention options, which would you choose? 8 / 9

20% (20 per 100)

$100

Low

No

intervention_Choice8

Select

75% (75 per 100)

$25

High

No

intervention_Choice8

Select

**Chance that treatment will prevent myeloma completely**

**Monthly out of pocket cost**

**Personal inconvenience**

**Risk of long- term side effects?**

intervention_Choice9

If these were your only treatment/intervention options, which would you choose? 9 / 9

20% (20 per 100)

$100

Low

Yes

intervention_Choice9

Select

50% (50 per 100)

$500

Low

No

intervention_Choice9

Select

**Chance that treatment will prevent myeloma completely**

**Monthly out of pocket cost**

**Personal inconvenience**

**Risk of long- term side e!ects?**

**REFERENCES**

1. El-Khoury H, Lee DJ, Alberge JB, et al. Prevalence of monoclonal gammopathies and clinical outcomes in a high-risk US population screened by mass spectrometry: a multicentre cohort study. *Lancet Haematol*. May 2022;9(5):e340-e349. doi:10.1016/S2352-3026(22)00069-2

2. Gurmankin Levy A, Shea J, Williams SV, Quistberg A, Armstrong K. Measuring perceptions of breast cancer risk. *Cancer Epidemiol Biomarkers Prev*. Oct 2006;15(10):1893-8. doi:10.1158/1055-9965.EPI-05-0482

3. Fagerlin A, Zikmund-Fisher BJ, Ubel PA, Jankovic A, Derry HA, Smith DM. Measuring numeracy without a Math test: Development of the subjective numeracy scale. *Medical Decision Making*. Sep-Oct 2007;27(5):672-680. doi:Doi 10.1177/0272989x07304449

4. Carleton RN, Norton MA, Asmundson GJ. Fearing the unknown: a short version of the Intolerance of Uncertainty Scale. *J Anxiety Disord*. 2007;21(1):105-17. doi:10.1016/j.janxdis.2006.03.014
